# Collagenous Colitis in a Patient With Gastric Cancer Who Underwent Chemotherapy

**DOI:** 10.7759/cureus.39466

**Published:** 2023-05-25

**Authors:** Masaya Iwamuro, Takehiro Tanaka, Shunsuke Kagawa, Shoko Inoo, Motoyuki Otsuka

**Affiliations:** 1 Department of Gastroenterology and Hepatology, Okayama University Hospital, Okayama, JPN; 2 Department of Pathology, Okayama University Hospital, Okayama, JPN; 3 Department of Gastroenterological Surgery, Okayama University Graduate School of Medicine, Dentistry, and Pharmaceutical Sciences, Okayama, JPN; 4 Department of Gastroenterology and Hepatology, Okayama University Graduate School of Medicine, Dentistry, and Pharmaceutical Sciences, Okayama, JPN

**Keywords:** immune checkpoint inhibitor, chemotherapy-induced diarrhea, immune-related adverse event colitis, colonoscopy, collagenous colitis

## Abstract

Herein, we present a case of collagenous colitis in a patient who underwent chemotherapy for gastric cancer, comprising five cycles of S-1 plus oxaliplatin and trastuzumab, followed by five cycles of paclitaxel and ramucirumab and seven cycles of nivolumab. The subsequent initiation of trastuzumab deruxtecan chemotherapy led to the development of grade 3 diarrhea after the second cycle of treatment. Collagenous colitis was diagnosed via colonoscopy and biopsy. The patient’s diarrhea improved following the cessation of lansoprazole. This case highlights the importance of considering collagenous colitis as a differential diagnosis, in addition to chemotherapy-induced colitis and immune-related adverse event (irAE) colitis, in patients with similar clinical presentations.

## Introduction

Collagenous colitis, a subtype of microscopic colitis, manifests as a persistent gastrointestinal condition characterized by symptoms of chronic diarrhea, abdominal pain, cramping, and bloating [1-3]. This disorder is typified by the accumulation of a dense layer of type I and III collagen below the epithelial cells of the colon [1,4]. Notably, there exists a correlation between the onset of collagenous colitis and the use of proton pump inhibitors, particularly lansoprazole [5]. Due to its microscopic nature, this condition often lacks macroscopic abnormalities during colonoscopic examination and necessitates an endoscopic biopsy for diagnosis.

Chemotherapeutic agents can also cause undesirable inflammation of the colon, which manifests as chemotherapy-induced diarrhea [6,7]. Chemotherapy-induced diarrhea can cause additional symptoms, such as abdominal discomfort and bloody stools [7]. The pathogenesis of this disease is linked to chemotherapy-induced injury of the mucosal lining of the colon, culminating in a cascade of inflammation and ulceration of the colorectal region. In addition, as immune checkpoint inhibitors have emerged as an established therapeutic option for malignant neoplasms, attention has been drawn to immune-related adverse events (irAEs) [8-11]. irAEs affect various systemic organs, with the gastrointestinal tract having the highest incidence [12]. The incidence of irAEs within the gastrointestinal tract varies depending on the specific immune checkpoint inhibitor employed. Notably, studies have reported diarrhea in 27%-54% of cancer patients undergoing cytotoxic T-lymphocyte-associated antigen-4 (CTLA-4) inhibitor therapy, while colitis occurred in 8%-22% of these patients [12]. Given the severity of irAEs that necessitates immunomodulatory interventions, including corticosteroids and biologics, it is imperative to promptly recognize and manage this condition.

Herein, we present the case of a patient who experienced diarrhea while undergoing chemotherapy for gastric cancer. Despite the initial suspicion of chemotherapy-induced diarrhea or irAE colitis, an endoscopic biopsy revealed a thickened collagen band beneath the surface epithelium, indicating a diagnosis of collagenous colitis. The patient’s diarrheal symptoms ameliorated subsequent to the discontinuation of lansoprazole. This case underscores the importance of endoscopic and microscopic evaluations in patients who exhibit diarrhea while receiving chemotherapy and/or immune checkpoint inhibitor therapy.

## Case presentation

A 67-year-old Japanese male underwent proximal gastrectomy for gastric cancer. He had no other underlying diseases except gastric cancer. The postsurgical pathological analysis confirmed the presence of signet ring cell carcinoma, which measured 7 mm and was restricted to the mucosal layer (pT1a) without lymphovascular invasion. Thus, it was concluded that the curative resection of the gastric cancer had been successful. Postoperative surveillance for recurrent and/or metachronous disease was carried out for five years. No examinations were performed after the patient turned 72 years as no recurrence of gastric cancer was observed during this period. However, he experienced tarry stool at the age of 77 years, and subsequent esophagogastroduodenoscopy revealed the presence of gastric cancer in the antrum of the remnant stomach. The patient underwent total gastrectomy, and the pathological analysis of the resected specimen led to a diagnosis of gastric cancer invading the submucosal layer and lymph node metastasis. The human epidermal growth factor receptor-2 (HER2) status was defined as positive (3+) on immunohistochemistry.

Four months postoperatively, computed tomography and positron emission tomography revealed metastatic lesions in the liver. The patient received chemotherapy, comprising five cycles of S-1 (100 mg/day) plus oxaliplatin (150 mg) and trastuzumab (300 mg), followed by five cycles of paclitaxel (127 mg) and ramucirumab (432 mg) and seven cycles of nivolumab (240 mg). Each treatment regimen was altered to a different course of therapy due to tumor progression. Subsequently, trastuzumab deruxtecan (300 mg) chemotherapy was initiated. Grade 1 diarrhea (defined as an increase of less than four stools per day over baseline) and grade 2 anorexia (defined as altered oral intake without significant weight loss or malnutrition) developed three days after the initial treatment with trastuzumab deruxtecan. Since his diarrhea was exacerbated to grade 3 (defined as an increase of more than seven stools per day over baseline) after the second cycle of trastuzumab deruxtecan treatment, chemotherapy was suspended. His stool was observed to be nonfatty, non-bloody, and watery. The patient was referred to the department of gastroenterology for further treatment of the diarrhea. At this point, he had been taking *Lactobacillus* preparations, aluminum silicate, albumin tannate, loperamide, and fexofenadine for diarrhea; domperidone for anorexia; branched-chain amino acid preparations for hypoalbuminemia; azosemide for edema; and etizolam for insomnia. Furthermore, to alleviate symptoms of heartburn, lansoprazole was prescribed 17 months prior, and the patient had been consistently taking a daily dosage of 15 mg of lansoprazole.

**Figure 1:**
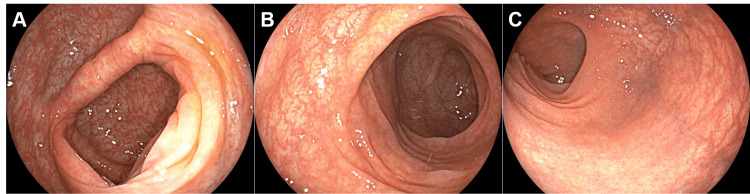
Colonoscopy images. Colonoscopy demonstrated an absence of discernible inflammation in the colorectal mucosa (A, cecum; B, transverse colon; C, rectum).

Blood tests (Table 1), computed tomography, esophagogastroduodenoscopy, and colonoscopy were performed to investigate the cause of diarrhea. He tested negative for cytomegalovirus antigenemia in his blood and for *Clostridium difficile* toxins in his stool. Stool testing results for norovirus and rotavirus were negative, and no pathogenic organisms were detected in the stool culture. The increase in carcinoembryonic antigen levels to 6,496 ng/mL was considered a result of the advancement of gastric cancer, characterized by the presence of multiple metastases. Although ascites and metastatic liver tumor enlargement were observed on computed tomography, no intestinal abnormalities were detected. Esophagogastroduodenoscopy revealed intact esophageal and jejunal mucosa and anastomotic sites. Colonoscopy revealed the absence of apparent inflammation in the colorectal mucosa (Figure 1).

**Table 1:** Significant laboratory results on presentation.

Blood test results (units)	Patient value	Reference range
White blood cells (/μL)	6,000	3,300-8,600
Neutrophil (%)	80.4	40-70
Hemoglobin (g/dL)	10.2	11.6-14.8
Platelets (/μL)	15.8×10^4^	15.8×10^4^-34.8×10^4^
Total protein (g/dL)	5.4	6.6-8.1
Albumin (g/dL)	2.3	4.1-5.1
Creatinine (mg/dL)	0.67	0.46-0.79
Lactate dehydrogenase (U/L)	200	124-222
Sodium (mmol/L)	143	138-145
Potassium (mmol/L)	3	3.6-4.8
Calcium (mmol/L)	8	8.8-10.1
Aspartate aminotransferase (U/L)	41	13-30
Alanine aminotransferase (U/L)	25	7-23
γ-Glutamyl transpeptidase (U/L)	100	9-32
C-reactive protein (mg/dL)	1.69	0-0.15
Carcinoembryonic antigen (ng/mL)	6,496	0-5
Cancer antigen 125 (U/mL)	222	0-55

**Figure 2:**
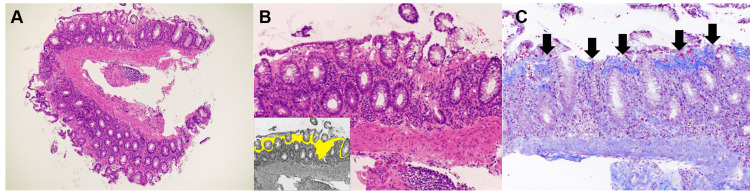
Pathological images of the biopsy specimens obtained from the colon. A collagen band measuring >40 µm in thickness was observed beneath the surface epithelial cells (hematoxylin and eosin stain: A, ×4; B, ×10). The inserted image depicts a collagen band, which is presented as a yellow area (B). Masson’s trichrome stain showed a collagen band as a bluish deposition (C: ×10, arrows).

**Figure 3:**
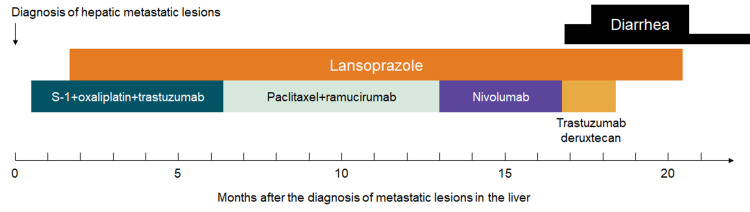
Chronological representation delineating the patient’s episodes of diarrhea and the administered pharmacological interventions.

However, biopsy specimens obtained from the cecum, transverse colon, sigmoid colon, and rectum showed a thickened collagen band measuring over 40 µm in thickness beneath the surface epithelial cells (Figure 2). Hence, although chemotherapy-induced diarrhea and irAE colitis were regarded as potential differential diagnoses, collagenous colitis was diagnosed through pathological analysis. The patient’s diarrhea improved to grade 1 status one week following the discontinuation of lansoprazole (Figure 3).

## Discussion

The diagnosis of collagenous colitis is generally made by identifying the presence of a subepithelial collagen band exceeding 10 µm in thickness, in combination with an inflammatory infiltrate located in the lamina propria [13,14]. According to a pooled analysis, the incidence rate of collagenous colitis was estimated at 4.9 cases per 100,000 person-years, with a 95% confidence interval ranging from 4.2 to 5.7 [13,14]. Although the exact cause of collagenous colitis has not been fully elucidated, several factors are known to increase the risk of developing the condition, including the age of >50 years, female sex, genetics, autoimmune disorders such as rheumatoid arthritis and lupus, and medications such as nonsteroidal anti-inflammatory drugs and proton pump inhibitors [13]. Particularly, some research has suggested that the long-term use of proton pump inhibitors causes collagenous colitis, probably due to changes in the gut microbiome or alterations in the immune system that can occur as a result of administering proton pump inhibitors [15,16]. In the present case, the patient was administered lansoprazole, and the discontinuation of this medication led to the resolution of diarrhea, implying that lansoprazole may have been implicated in the development of collagenous colitis.

As described above, it is imperative to consider the possibility of chemotherapy-induced diarrhea and irAE colitis as the etiologies of diarrhea in patients who currently receive or have previously received chemotherapy and/or immune checkpoint inhibitor therapy. In the current patient, diarrhea manifested three days after the initial administration of trastuzumab deruxtecan, composed of a humanized anti-HER2 antibody and a topoisomerase I inhibitor. The literature suggests that diarrhea is the predominant gastrointestinal toxicity attributed to HER2 receptor blockade by targeted therapeutic agents [17,18]. The patient also received therapeutic intervention through seven cycles of nivolumab, which is an immune checkpoint inhibitor that hinders programmed cell death protein 1 (PD-1) located on the surface of immune cells. This augments the ability of the immune system to identify and attack cancer cells with greater efficacy. Concurrently, immune checkpoint inhibitors disrupt the regulatory pathways of the immune system and can damage multiple organs and systems within the body, such as the skin, gastrointestinal tract, liver, endocrine glands, and lungs. Wang et al. conducted a meta-analysis wherein they demonstrated that patients receiving PD-1 signaling inhibitors had an overall incidence of irAEs of 26.8% (95% confidence interval: 21.7-32.6) [19]. Notably, they identified diarrhea as the most prevalent irAE in patients who received nivolumab treatment, with an incidence of approximately 10%-13%. In the present patient, irAE colitis was excluded because of the absence of specific pathological features, such as basal lymphoplasmacytosis, crypt architectural irregularity, crypt abscesses, and apoptosis [20].

The exacerbation of preexisting collagenous colitis after nivolumab exposure is another conceivable cause of diarrhea in the current patient. Thomas et al. retrospectively investigated 10 patients with microscopic colitis (six with collagenous colitis and four with lymphocytic colitis) who received PD-1 or programmed cell death ligand 1 inhibitors [21]. The authors reported that eight patients experienced exacerbations of colitis after immune checkpoint inhibitor therapy, which necessitated immunosuppressive treatment. Although its incidence is low, other cases of collagenous colitis in patients undergoing immune checkpoint inhibitor therapy have been reported [22-24]. Currently, the mechanisms and prevalence of collagenous colitis exacerbation following immune checkpoint inhibitor therapy remain unclear, owing to the rarity of this disease. In the present case, even though the patient was not receiving immunotherapy at the time, we cannot exclude the possibility that previous exposure to nivolumab resulted in the development or aggravation of colitis. Furthermore, it is crucial to recognize that the potential implication of trastuzumab deruxtecan in the pathogenesis of colitis cannot be overlooked, as evident from the occurrence of diarrhea subsequent to the administration of trastuzumab deruxtecan. Additional investigations are warranted to elucidate the influence of immune checkpoint inhibitors and/or chemotherapeutic agents on the pathophysiology of collagenous colitis.

## Conclusions

We presented a case of collagenous colitis that developed during systemic chemotherapy for advanced gastric cancer. This case highlights the importance of colonoscopy and histological examination of endoscopically acquired biopsy specimens for the diagnosis of this disease, as well as for differentiating it from chemotherapy-induced diarrhea and irAE colitis, even though it occurs infrequently.
